# Identification and immunological characterization of cuproptosis-related molecular clusters in ulcerative colitis

**DOI:** 10.1186/s12876-023-02831-2

**Published:** 2023-06-27

**Authors:** Yunfei Pu, Xianzhi Meng, Zhichen Zou

**Affiliations:** 1 The First Affiliated Hospital of Harbin Medical University, Harbin, Heilongjiang China; 2 Department of Minimally Invasive Biliary Surgery, The First Affiliated Hospital of Harbin Medical University, Harbin, 150000 Heilongjiang China

**Keywords:** Cuproptosis, Ulcerative colitis, Prediction model, Molecular clusters, Machine learning

## Abstract

**Background:**

Ulcerative colitis is one of the two main forms of inflammatory bowel disease. Cuproptosis is reported to be a novel mode of cell death.

**Methods:**

We examined clusters of cuproptosis related genes and immune cell infiltration molecules in 86 ulcerative colitis samples from the GSE179285 dataset. We identified the differentially expressed genes according to the clustering method, and the performance of the SVM model, the random forest model, the generalized linear model, and the limit gradient enhancement model were compared, and then the optimal machine model was selected. To assess the accuracy of the learning predictions, the nomogram and the calibration curve and decision curve analyses showed that the subtypes of ulcerative colitis have been accurately predicted.

**Results:**

Significant cuproptosis-related genes and immune response cells were detected between the ulcerative colitis and control groups. Two cuproptosis-associated molecular clusters were identified. Immune infiltration analysis indicated that different clusters exhibited significant heterogeneity. The immune scores for Cluster2 were elevated. Both the residual error and root mean square error of the random forest machine model had clinical significance. There was a clear correlation between the differentially expressed genes in cluster 2 and the response of immune cells. The nomogram and the calibration curve and decision curve analyses showed that the subtypes of ulcerative colitis had sufficient accuracy.

**Conclusion:**

We examined the complex relationship between cuproptosis and ulcerative colitis in a systematic manner. To estimate the likelihood that each subtype of cuproptosis will occur in ulcerative colitis patients and their disease outcome, we developed a promising prediction model.

## Introduction

Ulcerative colitis (UC) was first discovered in 1859, and it is one of two main types of inflammatory bowel disease (IBD) [1]. It is a chronic immuno-mediated IBD, though its exact pathogenesis is not known. Prolonged inflammation will damage gastrointestinal function, such as causing abdominal pain, bleeding and other symptoms [2]. In addition, diagnosis of digestive system diseases and evaluation of the prognosis of patients through inflammatory indicators and infiltration levels of immune cells [3]. For example, Posul et al. 2015found that the ratio of neutrophils to lymphocytes could predict whether ulcerative colitis was in the active phase [4].

A number of factors have been linked to this condition, including dysregulated immune response, altered microflora in the gut, genetic susceptibility, and environmental factors [5]. Globally, the prevalence is high in developed countries, and is rapidly increasing in newly industrialized countries [6, 7]. Conley et al., 2017 demonstrated by 2025, the population is expected to reach 30 million worldwide [8]. Healthcare and social costs are significant as a result of the excessive morbidity and mortality rate caused by UC [9–11]. Abraham and Kane., 2012 demonstrated in spite of having the same phenotype, extreme early-onset IBD may have a different pathophysiology from IBD of earlier onset [12, 13]. As IBD incidence increases in developing countries and phenotypes are similar between Asians and Westerners, ethnicity appears not to be an important factor in UC. Due to the phenotypic similarity of UC across the globe, environmental changes may also be responsible for the observed epidemiological trends. Most patients with UC suffer from hemorrhagic diarrhea, and the clinical presentation is heterogeneous. Only 30% and 15% of patients have aggressive or generalized colitis, respectively. Solberg et al., 2009 reported that half of the patients may not respond to pharmacological treatment, resulting in a more complicated disease course [14]. UC is therefore formed as a result of different etiologies, which influence the course and severity of subsequent illness [15–17]. It is unfortunate that UC lacks a satisfactory treatment due to its clinical heterogeneity and complexity of pathological types. Despite the increasing association of biomarkers with UC, these results aren't convincing. In order to enhance clinical credibility, we extended the study of UC to the molecular subtype.

Researchers have previously attempted to define heterogeneity and treat UC [18]. Copper ion homeostasis in the human body is maintained by copper absorption, transport, and excretion, according to a recent study. Tang et al., 2022 reported that copper was the cause of a new form of cell death, called copperocytosis, which was related to the tricarboxylic acid cycle metabolism disorder [19, 20]. This mode of death is not completely independent of other regulatory modes of death, suggesting a close connection. Increasing evidence suggests that unbalanced copper homeostasis can affect tumor growth and induce tumor cell death. Related studies suggest that inhibition of mitochondrial pyruvate carrier and electron transport chain activity can mitigate the damage caused by cuproptosis [21–23]. Moreover, the infiltration of multiple immune cells in inflammatory diseases may be regulated through cuproptosis. Zhao et al., 2022 suggested that copper death led to excessive survival or proliferation of multiple immune cells in the synovial tissue of rheumatoid arthritis [24]. While copper plays a vital role in tumor immunity and cancer therapy, the induction of cell death in non-tumor is less well studied. In order to provide more personalized treatment for UC, it is imperative to identify more appropriate molecular clusters. Various disease mechanisms have not been studied in depth for the mechanism of non-tumor cuproptosis. Thus, cuproptosis genetic characteristics were used to identify UC subtypes.

This study was the first to systematically examine the differential expression and immune signature of cuproptosis-related genes in normal and UC individuals. Using 19 cuproptosis-related genes (CRGs) expression profiles, we divided 86 UC patients among 254 IBD patients into two groups, and found differences in immune cells between the two groups. By using the WGCNA algorithm, the differentially expressed genes (DEGs) between them, and the functional pathways of the differentially expressed genes were further explained. Additionally, we compared multiple machine learning algorithms to construct a special learning prediction model that presents different molecular clusters of UC-associated patients. Validation of the predictive model was conducted using decision curve analysis, nomographic analysis and calibration curve analysis. And there are two random forest models for further verification.

## Materials

### Preprocessing and acquisition of data

Related microarray data sets are obtained from GEO( www.ncbi.nlm.nih.gov/geo) including one experimental group GSE179285 data set, two verification groups GSE107597 data set and GSE92415 data set [25]. There were 31 healthy samples and 55 UC colon tissue samples in the trial GSE179285 datasets (GPL6480 platform), while the validation group GSE107597 datasets (GPL15207 platform), which included rectal tissue from 44 normal subjects and 75 UC (13–56 years old) samples, and the GSE92415 datasets (GPL13158 platform), including 21 normal and 162 UC (19–77 years old) colon tissue samples. Once the samples were downloaded, these raw data were collated and summarized in the next step. We used the Robust Multiarray Average (RMA) method ("affy" R package). The resulting expression measurements of the corresponding gene were estimated by RMA for all probes of the gene. The model generated by this algorithm will produce an estimate of the gene signal that takes into account the probe effect.

### The infiltration of immune cells was analyzed

We use CIBERSORT algorithm (https://cibersort.stanford.edu/) and signature matrix of gene expression data to analyze 22 kinds of immune cells corresponding relations, and *P* values were calculated by the CIBERSORT for each. We considered immune cell fractions accurate when they had *P* < 0.05. The total number of the 22 immune cells per sample is identical [26].

### CRG and immune cell infiltration correlation analysis

We analyzed the percentage relationship between CRG and immune cell expression, in order to further demonstrate the link between CRGs and UC-related immune cell properties. *P* < 0.05 indicates relevant research significance, according to the spearman correlation coefficient. Lastly, plot results used the "corrplot" R package (version 0.92).

### Patients with UC are clustered unsupervised

As a result of the previous report, a total of 12 CRGs were obtained [19, 27]. We used the K-means algorithm for 1,000 iterations and the consensus cluster package for unsupervised cluster analysis to classify 55 UC samples into different clusters. The consensus matrix, consistent cluster score (> 0.9) and cumulative distribution function (CDF) curve were evaluated to select the optimal number of clusters, and the maximum number of clusters was K = 9.

### Gene set variation analysis (GSVA) analysis

In order to show the differences of gene sets among different CRGs clusters,The method of Wu et al. 2021 reported that the R package of "GSVA" (version 2.11) was used for analysis [28]. For further GSVA analysis, we used the database known to be symbols from the MSigDB website "c2.cp.kegg.symbols" and "c5.go. Symbols". By comparing GSVA scores between different CRG clusters, we used the "limma" R package (version 3.52.1) to evaluate the score, where a score greater than 2 indicates that the study is meaningful.

### Weighted gene co-expression network analysis (WGCNA)

We used the R package “WGCNA” (version 1,700.3) to identify co-expression modules [29]. In WGCNA analysis, 25% of the genes with the strongest correlation were selected for accuracy analysis. Firstly, a weighted adjacency matrix is set up using the optimal soft power, and then the matrix is transformed into a topological overlapping matrix (TOM). The next step is to analyze the module obtained when the minimum module value of the TOM dissimilarity of the hierarchical clustering tree algorithm is 100. The characteristic genes in each module represent overall gene expression in that module, and each module has a random color. The module significance (MS) index is what we need, which shows the relationship between a specific module and the disease. The term gene significance (GS) is used to describe the association between a gene and a clinical phenotype.

### Multiple machine learning methods are used to construct a predictive model

Random forest model (RF), support vector machine model (SVM), generalized linear model (GLM), and extreme gradient enhancement (XGB) are the machine learning models we built on different clusters of CRGs. RF is a prediction and regression technique that uses randomization and substitution to represent the technique's precise predictions [30]. SVM can describe the value of our predictions as a learning model to improve the value of our predictions [31]. GLM consists of linear components and link functions, which can correctly evaluate the relationship between independent or mutually characteristic normal distributions [32]. The XGB has a predictable and enhanced set of numbers, increasing the accuracy of our prediction models. DEGs specific to each cluster were chosen as explanatory variables, and the corresponding variables are different clusters. We randomly classified the 55 UC samples into two training and validation sets (55%, *N* = 30). Through grid search, the insertion symbol package automatically adjusts the parameters in these machine learning models. All these models were evaluated via fivefold cross validation. To interpret and visualize the distribution of residuals and the importance of features between the aforementioned four machine learning models, the "DALEX" package (version 2.4.0) was used. Use the "pROC" R package (version 1.18.0) to visualize the area under the ROC curve [33]. Thus, the top five variables that were associated with UC were determined through a machine learning model. Liu et al. 2022 reported that a Nomogram was created through the "nomogramEx" R package [34].

### Analyses of independent validation

Finally, the two validation group data sets GSE107597 and GSE92415 were further analyzed using the ROC curve of the "pROC" R package to evaluate the UC and no-UC predictions that we validated our experimental group model.

## Results

### Patients with UC have dysregulated cuproptosis regulators and activated immune responses

**Fig. 1:**
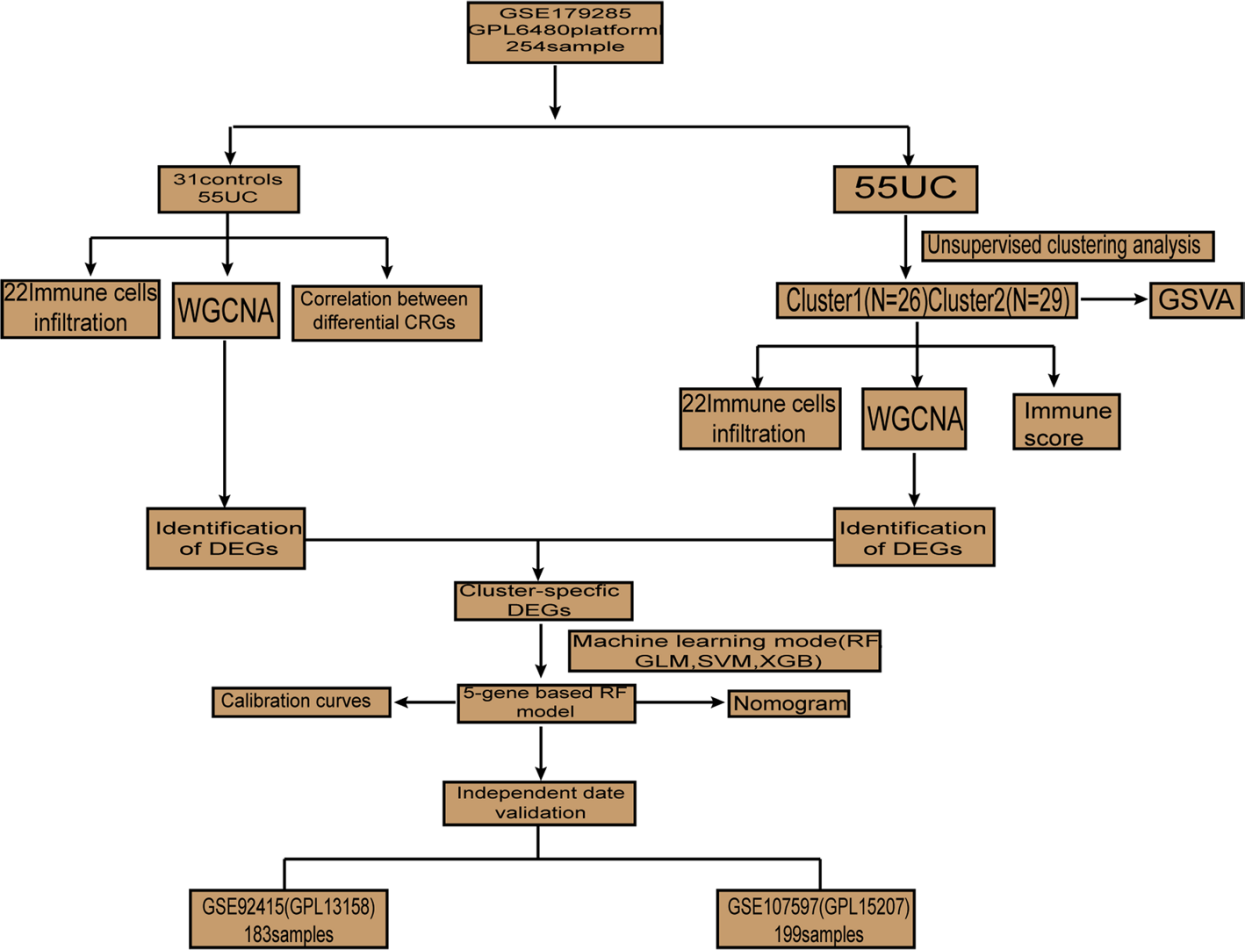
The flow-process diagram

**Fig. 2:**
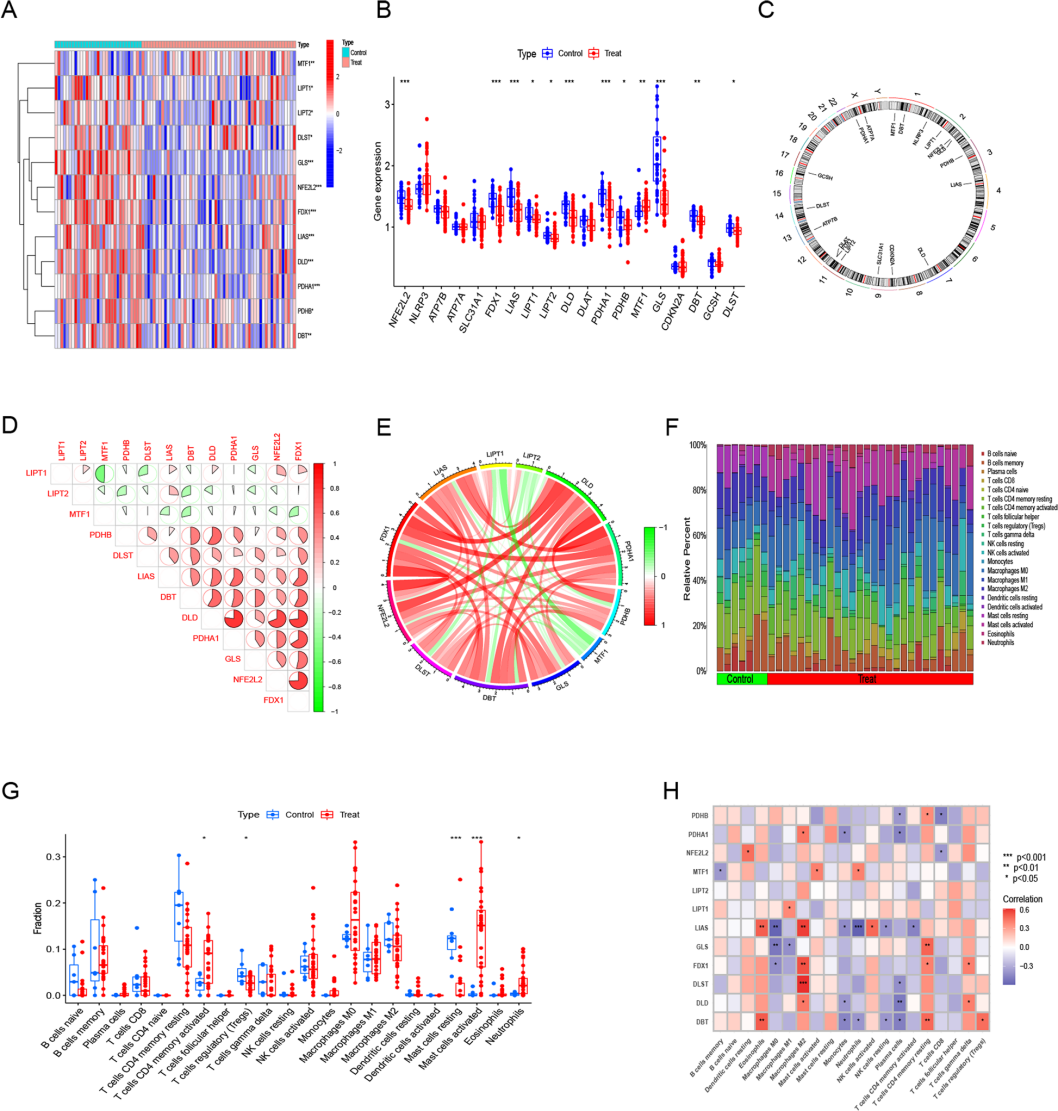
Differential expression of the CRGs in UC patients, heatmap of the expression correlation of the 12 CRGs **A**. Boxplots showed the expression of 12 CRGs between UC and no-UC, * *p* < 0.05, ** *p* < 0.01, *** *p* < 0.001 **B**. The position of the 12 CRGs on the chromosome was shown **C**, correlation analysis the 12 CRGs using the area of the pie chart **D**. Network diagram of the relationships of the 12 genes in the CRGs **E**. Showed the richness of 22 immune cell infiltration between UC and no-UC **F**. Boxplots showed differences between UC and no-UC, * *p* < 0.05, *** *p* < 0.001 **G**. Correlation analysis of immune cell infiltration in 12 CRGs **H**

To clarify the biological functions of cuproptosis regulators in the occurrence and progression of UC. A detailed flow chart of the study process was shown in Fig. 1. We identified 12 CRGs as differentially expressed cuproptosis genes in the evaluation prediction model. These include DLST, DBT, GLS, FDX1, MTF1, NFE2L2, PDHA1, DLD, LIPT2, LIPT1, LIAS, PDHB expression levels that are higher than those in non-UC controls (Figs. 2A–C). Afterward, because we wanted to determine whether the cuproptosis-related regulatory genes have some clinical significance in UC patients, a correlation analysis was done to study the differential expression of CRGs (Fig. 2D). Surprisingly, there was a strong synergistic effect between some cuproptosis modulators, such as PDHA1 and DLD. Conversely, there is also some antagonism between LIPT 1 and MTF 1. Further study found that DBT and DLD were closely related to different regulators. In addition, a circle graph of a gene perfectly shows the differential graph of CRGs (Fig. 2E).

According to the CIBERSORT algorithm, we are shown the proportion of 22 immune cells, and we determine the difference in the immune system in UC and non-UC (Fig. 2F). As a result, UC patients presented higher levels of infiltrating CD4T cells, mast cells that were resting, mast cells that were activated, and neutrophils (Fig. 2G), indicating that UC may be caused by immune system alternations. Macrophage M2 showed a positive relationship with epidermal growth regulators (Fig. 2H). The CRGs appear to play an important role in both the immune infiltration of UC as well as its molecular regulation.

### The identification of clusters of cuproptosis in UC

**Fig. 3:**
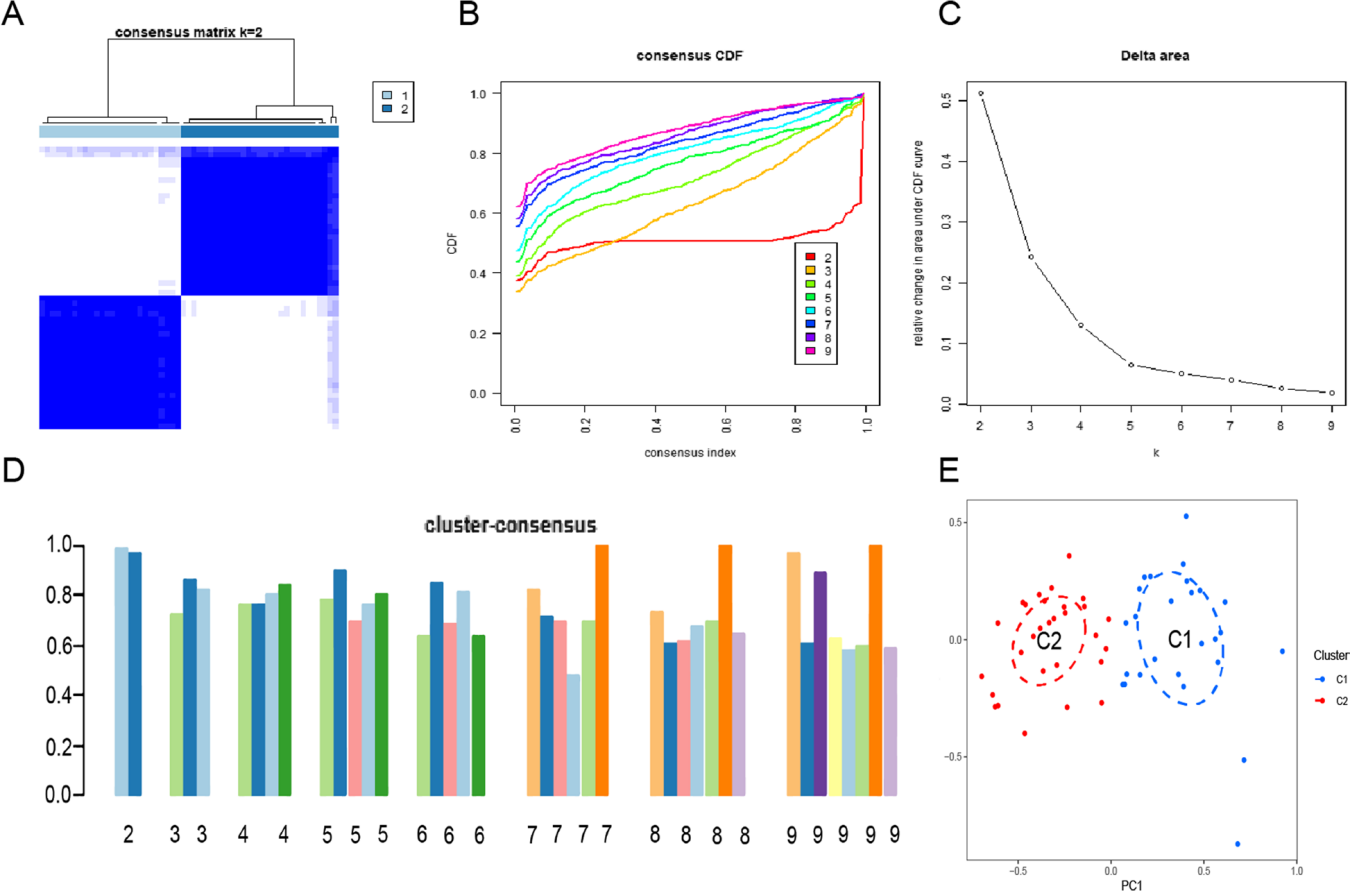
For the identification of cuproptosis-related molecular clusters in UC, a consensus clustering matrix with k = 2 was chosen and was the best molecular cluster classification **A**. CDF delta area curves **B**. Scores for consensus clustering **C**. A heatmap of non-negative matrix **D**. A PCA analysis of the distribution of the two molecular clusters **E**,

We used a consensus clustering algorithm to group 55 UC samples by genes of 12 CRGs to identify cuproptosis-related expression patterns. When k = 2, cluster numbers were most stable, and CDF curves ranged between 0.2 and 0.6 (Figs. 3A,B). The CDF curve shows the degree of difference in correlation between different k, while k has a minimum value of 2 and a maximum value of 9 (Fig. 3C). Moreover, Because the consistency score for each subtype is greater than 0.9, a k of 2 is the value of our best chosen molecular cluster (Fig. 3D). Based on the Principal Component Analysis (PCA) analysis, these two clusters differed significantly (Fig. 3E).

### Comparing cuproptosis clusters based on epidermal cell-related factors, immune cell infiltration characteristics, and pathway annotation

**Fig. 4:**
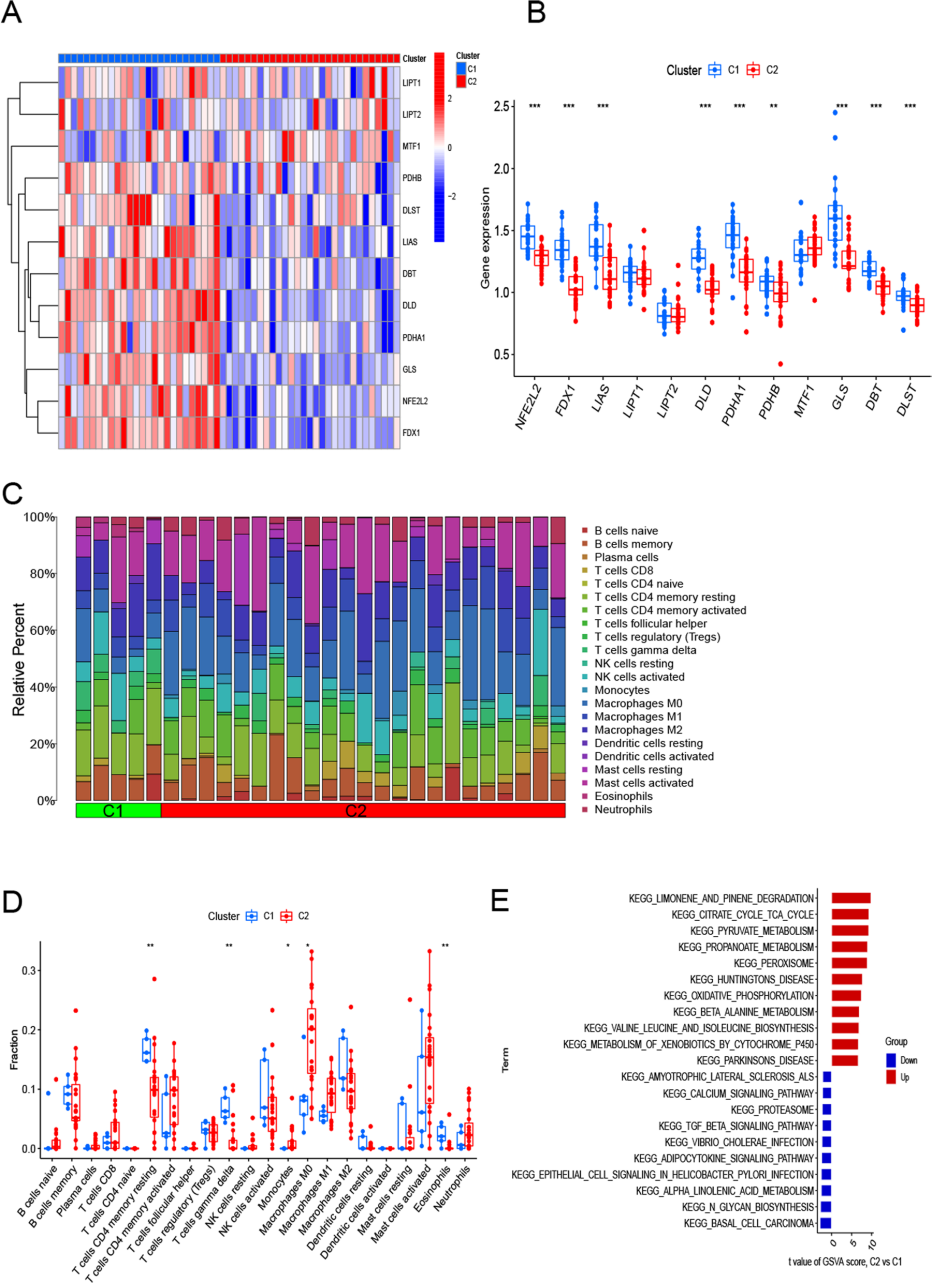
It showed the molecular characteristics and immune characteristics between the two molecular clusters associated with cuproptosis. Heatmap of the expression signature between the 12 CRGs and the two molecular clusters **A**. Boxplot exhibited the expression of the 12 CRGs between the two molecular clusters, **p < 0.01, ***p < 0.001 **B**. Differences in richness of 22 immune cells in the two molecular clusters **C**. Boxplots showed the infiltration richness of the immune cells between the two molecular clusters * *p* < 0.05, ** *p* < 0.01 **D**. There are some differences in the hallmark pathway activities between the two molecular clusters ordered by the different t-value of the GSVA method **E**

Our first step was to assess the differential expression of the 12CRGs between Cluster1 and Cluster2 to determine their molecular characteristics. Both cuproptosis patterns exhibit distinct expression landscapes of CRGs (Fig. 4A). Cluster 1 of cuproptosis exhibited high expression levels of PDHA1, NFE2L2, FDX1, LIAS, DLD, PDHB, DBT, GLS, and DLST (Fig. 4B). Further, we examined the immune-infiltrating cells and the immune environment between the two molecular clusters (Fig. 4C). While Cluster1 showed a greater proportion of resting and gamma delta T cells, Cluster2 showed a greater proportion of monocytes, macrophages M0, and mast cells activated (Fig. 4D). We used GSVA to determine the pathway differential expression levels of cluster-specific DEGs between Cluster1 and Cluster2. In Cluster2, oxidative phosphorylation, citrate cycle, TCA cycle, limonene, pinene degradation, peroxisome and metabolism signaling were enhanced, while Cluster1 had an upregulation of glycan biosynthesis, immune responses, cytokine receptors and Notch signaling activity. (Fig. 4E).

### Screening of gene modules and construction of co-expression networks

**Fig. 5:**
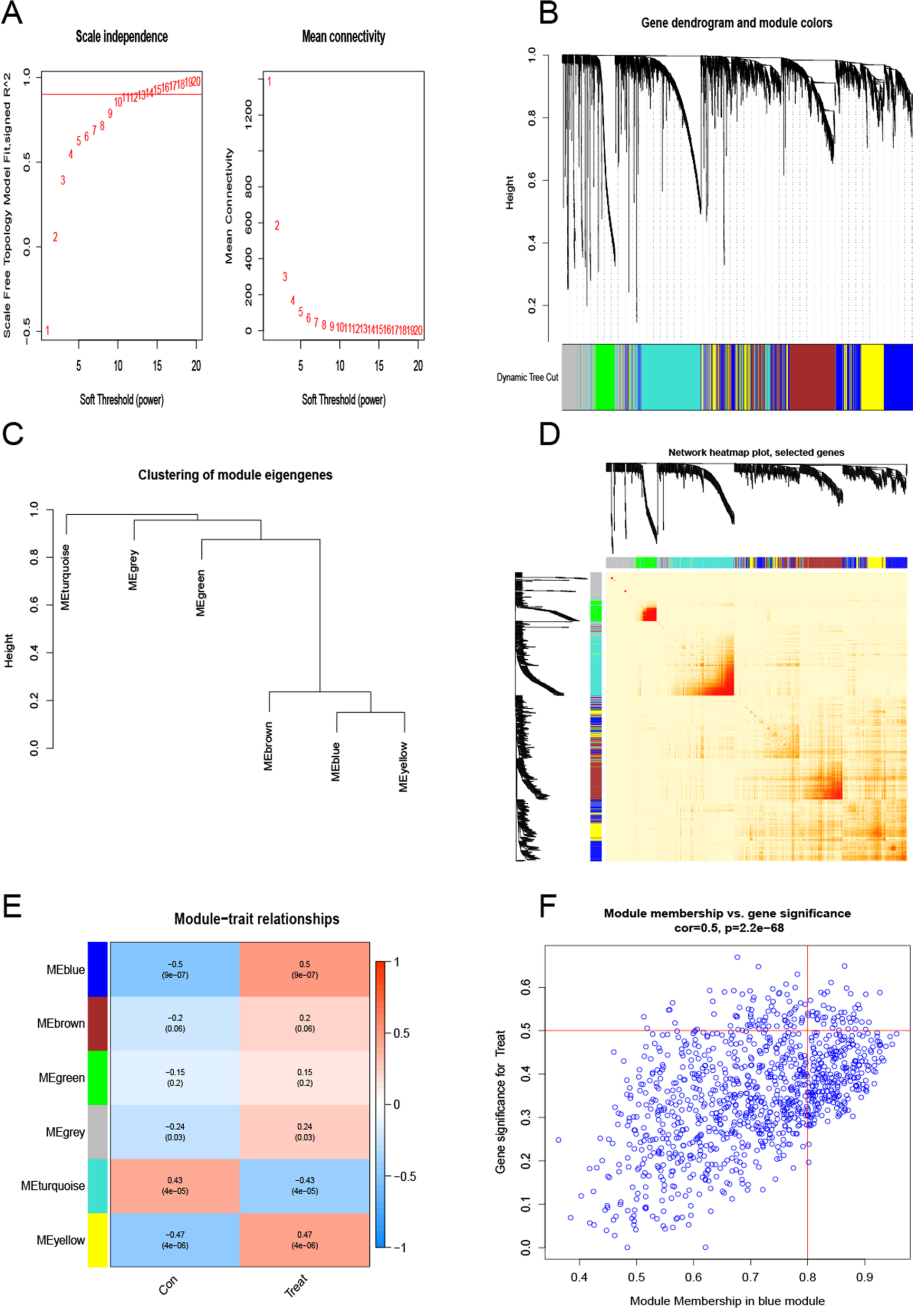
Gene co-expression network involved in gene expression in UC. Soft threshold power selection **A**. In the co-expression dendrogram, different colors represent different gene co-expression modules **B**. Showed the classification of gene module features **C**. Heatmap of the 6 genes representing the modules **D**. Rows represented modules, columns represented gene signature modules of clinical status and correlation analysis of clinical status **E**. Members of the module in blue were associated with genes in UC **F**

We created gene co-expression networks and modules, selected the strongest 25% of genes in the WGCNA analysis for accuracy results according to the gene expression variance of GSE179285, and set the scale-free R2 to 0.9 and soft power set to 11, to select the co-regulation modules of the required genes we need (Fig. 5A). Co-expression networks and modules were established using the WGCNA algorithm and also for the normal and UC subjects. A weighted adjacency matrix was constructed using the optimal soft power and transformed into TOM, where each module was randomly assigned a color, a total of 6 modules concerning coexpression of clinical features in the UC and no-UC studies (Figs. 5B–D). These genes in the 6 color modules were continuously applied for analyzing the similarity and adjacency of module-clinical features (UC and noUC) co-expression. The blue module is the most expressive, including 54 genes in the blue module (Fig. 5E). A weighted adjacency matrix was constructed using optimal soft power and transformed into a topological overlap matrix (TOM), where each module was randomly assigned a color. A total of 6 modules were co-expressed clinical features. Furthermore, blue modules are associated with module-related genes (Fig. 5F).

**Fig. 6:**
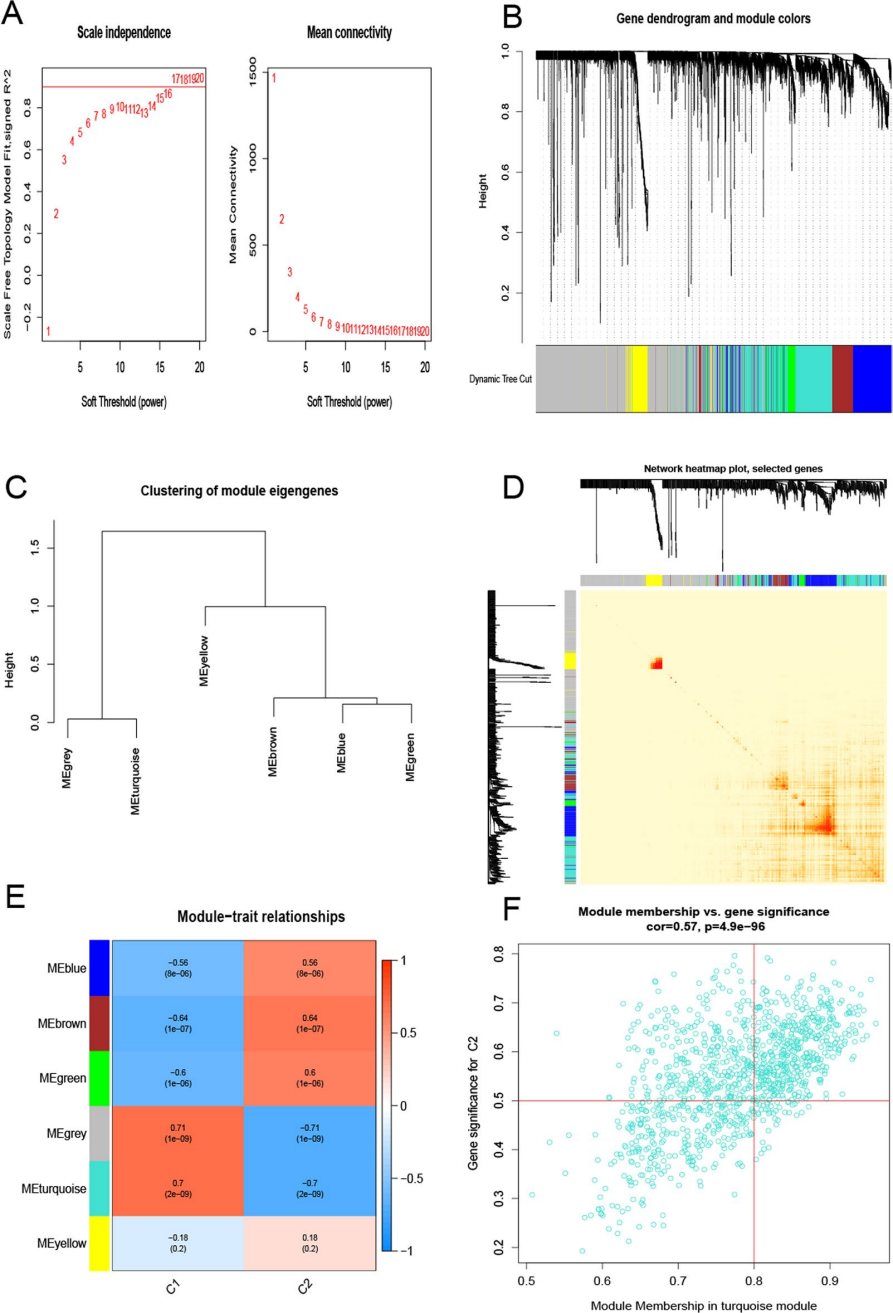
Co-expression network of expressed genes between the two cuproptosis-related molecular cluster. Soft threshold power selection **A**. In the co-expression dendrogram, different colors represent different gene co-expression modules **B**. Showed the classification of gene module features **C**. Heatmap of the 6 genes representing the modules **D**. Rows represented modules, columns represented gene signature modules of clinical status and correlation analysis of clinical status **E**. Scatter plot of gene correlation of members of the turquoise module with molecular cluster 2 **F**

Then we used the WGCNA algorithm to analyze the genes of the cuprotosis molecule clusters as β value of 16 and R 2 value of 0.9 (Fig. 6A), and also obtained 6 color modules containing a total of 4898 genes. The correlation heatmap concerning TOM also reveals significantly correlated genes (Figs. 6B–D). Between the two molecular clusters, in the relationship analysis of the relevant clinical characteristic modules, the UC cluster and the turquoise module (629 genes) were highly correlated (Fig. 6E). Correlation analysis showed that the turquoise color module gene was our best choice, and it had a significant correlation with the genes between the selected modules (Fig. 6F).

### Identifying cluster-specific DEGs

**Fig. 7:**
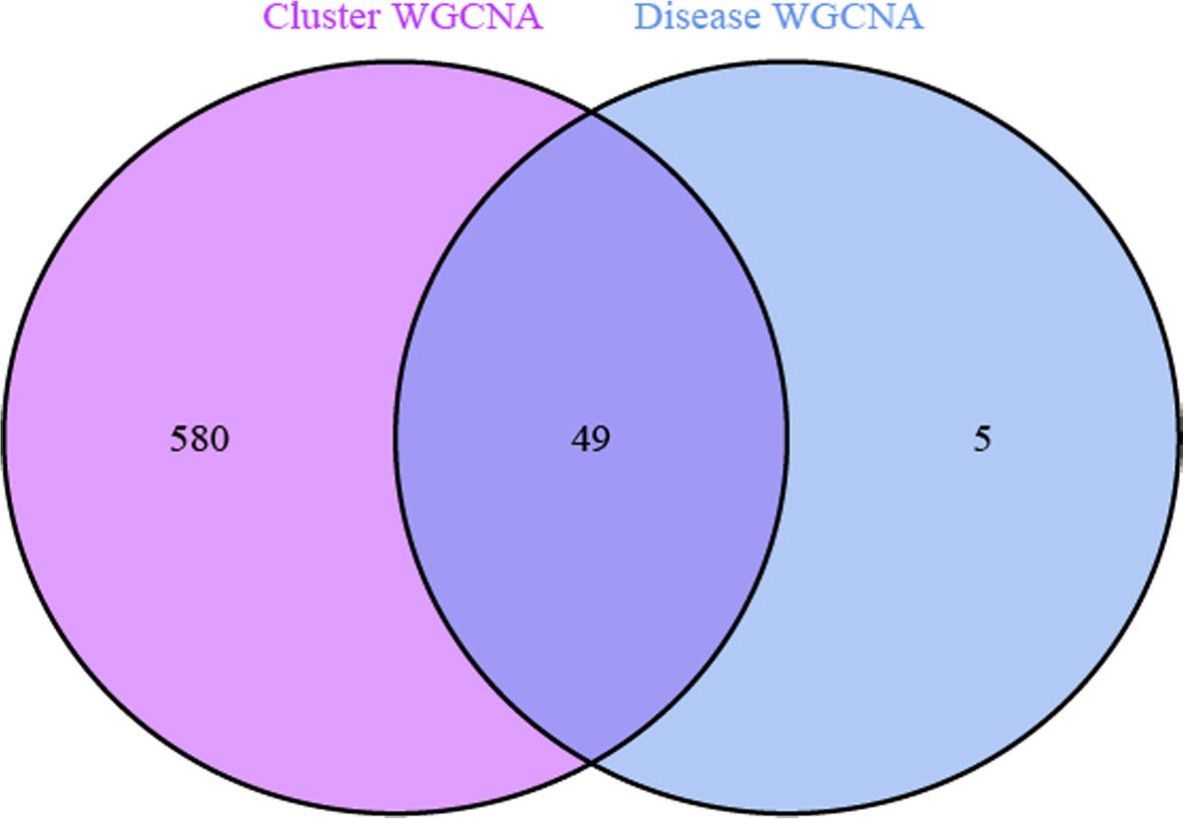
DEGs and biological characteristic differences between the two molecular clusters. Intersection between cuproptosis-related molecular cluster genes and module-related genes in the GSE179285 dataset

We performed analysis of the analyzed UC and noUC modules and obtained the associated crossover genes with 49 specific DEGs (Fig. 7).

### Machine learning model construction and assessment

**Fig. 8:**
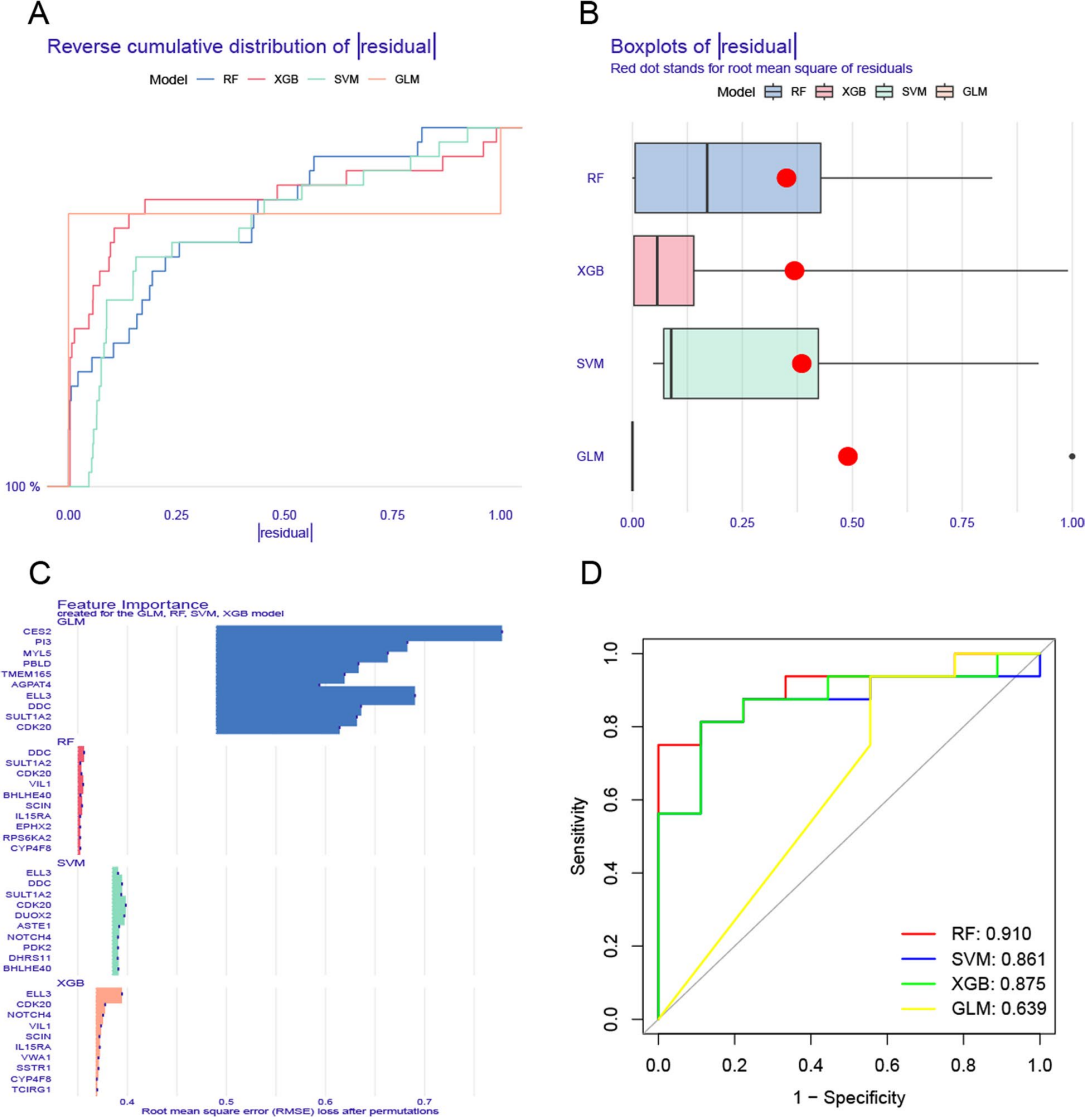
Construction and evaluation of the four machine models: RF, SVM, GLM, and XGB. Cumulative residual distribution of the four machine learning models **A**. Boxplots exhibited the magnitude of the residuals across the respective machine learning models, red dots indicated the root mean square of residuals (RMSE) **B**. Feature expression of the four machine-learning models **C**. The fivefold cross ROC analysis curve validates the RF, SVM, GLM, and XGB machine learning models **D**

Using the analysis to obtain the expression profiles of 49 cluster-specific DEGs, we used four machine learning models to obtain isoform genes called high predictive value, and these four learning models were RF, SVM, GLM, and XGB. The four models were constructed with the 'DALEX' package, and the residual expression distribution was plotted for each model. The RF learning model has the least residual expression (Figs. 8A,B). We selected the top 10 variables based on root mean square error (RMSE) (Fig. 8C). We obtained relevant ROC curves based on the five-fold cross-validation of the learning algorithm of the machine learning model, with AUC = 0.910 in RF, AUC 0.861 in SVM, AUC 0.639 in GLM, and AUC 0.875 in XGB (Fig. 8D).

**Fig. 9:**
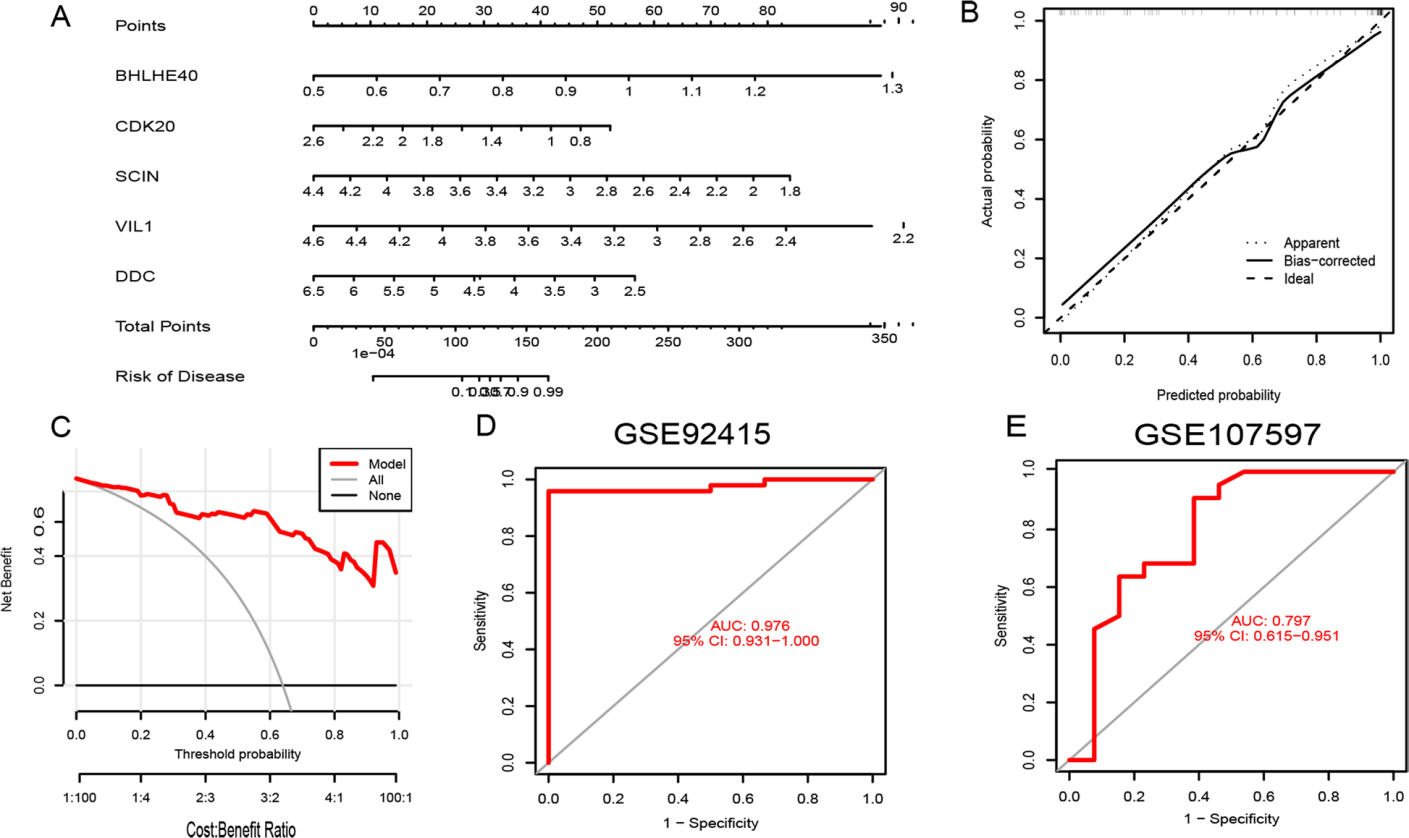
Further validation of the 5-gene-based RF model. Risk prediction of UC clusters used a nomogram of the 5-gene-based RF model **A**. To assess the predictive ability of the nomogram model, calibration curves **B** and DCA were made **C**. In the GSE92415 **D** and GSE107597 **E** datasets. ROC analysis used the final model genes based on fivefold cross-validation

According to the results of AUC, we chose RF as the model that shows the UC of different clusters. As a result of the RF model, the top five most important variables were selected as predictor genes (BHLHE40, CDK20, SCIN, VIL1, and DDC). The RF model was further evaluated by developing a nomogram to predict the crush disease group of the 86 patients with UC (Fig. 9A). We used the calibration curve and the decision curve to analyze the prediction value of the nomogram model, and obtained that the UC cluster prediction and the actual risk error are very small (Fig. 9B), and the decision curve analysis further proves the accuracy of our nomogram model (Fig. 9C). We then used two validation groups, the UC tissue database and the no-UC tissue database, to validate our 5-gene prediction model. In the GSE92415 dataset, the AUC value for the 5-gene prediction model was 0.976, while in the GSE107597 dataset, it was 0.797 (Figs. 9D,E).

## Discussion

We studied for the first time the expression of copper cuproptosis-related regulators in the colon tissues of UC and no-UC. At the same time, we also showed the specific role of cuproptosis-related gene phenotype and the immune microenvironment in UC. There were more dysregulated CRGs in UC patients than in normal individuals, suggesting that CRGs play an important role in UC. There were significant synergistic or antagonistic effects of some cuproptosis modulators in patients with UC, as evidenced by CRG interactions.

In this study, we found that there were higher infiltration levels in UC patients with T cells CD4 memory activated, mast cells activated, and neutrophils. UC is a common disease of the immune-mediated digestive system. The immune system influenced the disease development by modulating the infiltration of immune cells in the diseased area [35]. T cell immunotherapy had an outstanding advantage in the treatment of ulcerative colitis [36]. Furthermore, the important role of mast cells in resistance to bacterial infection and inflammation. The presence of microbial-gut-brain axis disease with mast cells as the main component in UC patients [37]. Neutrophils participated in disease progression through the secretion of proinflammatory cytokines and it was an abnormal infiltrating immune cell in UC [38].

Furthermore, we used unsupervised cluster analysis to analyze expression landscapes of CRGs in UC patients to identify two distinct cuproptosis-related clusters. The accuracy of our typing was further determined by PCA analysis. In Cluster1, immune scores were elevated and immune infiltration was relatively higher. The DEGs identified by Cluster 2 were primarily enriched in limonene and pinene degradation, citrate cycle, TCA cycle, and pyruvate metabolism processes, while The DEGs identified by Cluster 1 were primarily enriched in infection and immune-related pathways. For example,there were helicobacter pylori infection, vibrio cholerae infection, amyotrophic lateral sclerosis als. This indicates that having a higher degree of immune infiltration occurs in the Cluster1.

With the growing population and increasing databases, machine learning models of demographic and imaging indicators are increasingly used to analyze the clinic [39]. We therefore compared the predictive performance of the four machine learning models in UC, using the expression of the DEGs concerning UC, and selected RF as our optimal model. RF (AUC = 0.9829) indicates a high clinical significance for the machine learning model that we chose in the prediction study of UC. As a next step, we constructed an RF model based on five genes and five relevant variables (BHLHE40, CDK20, SCIN, VIL1, and DDC). Zhang et al., 2020 demonstrated that a gene called BHLHE40 was expressed exclusively in patients with UC, which acted as a crucial regulator of colon cancer cell growth. This suggestted that BHLHE40 might be a useful treatment for UC patients. However, its role in UC had not been studied or mentioned [40]. Lai et al., 2020 demonstrated that there was increasing research suggesting CDK20 was a key controlled of cell cycle checkpoints controlling cell proliferation and involved in the development of multiple cancers. He further speculated that this gene was a potential therapeutic target for inflammatory diseases, but it needed further verification [41, 42]. Chen et al., 2021 reported that PDE4D was crucial to the development of intestinal diseases by phosphorylating and activating the intestinal cell kinase.Given the high level of expression of SCIN in cancer tissues, we speculate that its expression level may also affect the diagnosis and prognosis of UC.Studies have found that SCIN also affects the colon's cellular immune infiltration. He did not do any further research [43, 44]. We further studied and improved the predictability for UC. Salewski et al., 2022 reported that VIL 1 involved in the tumor-associated immune infiltratio [45]. However, they did not mention the non-tumor UC patients, and we initially verified that colon inflammation was associated with the expression of VIL 1 protein. Artemaki et al., 2020 reported that positive correlation existed between DDC protein expression levels and unfavorable CRC prognoses [46]. We further verified the clinical correlation between DDC and UC.

Two validation data sets (AUC = 0.976 and 0.797) based on the 5-gene subject operating characteristic curve (ROC) provide new ideas for the diagnosis of UC prediction. In addition, we developed a nomogram model for diagnosing UC subtypes using BHLHE40, CDK20, SCIN, VIL1, and DDC. Based on the results of our study, we concluded that this prediction model is capable of making excellent predictions, indicating its value for clinical applications.

In this study, some limitations need to be highlighted. This paper is a full-coverage bioinformatics study for the analysis, so we need more clinical studies and experimental studies to validate the expression level of CRG involved in UC. More clinical datas and samples are required to support the accuracy of our model, the correlation between CRGs and immune infiltration.

In spite of the growing knowledge of the effects of environmental exposures, genetics, and gut microbes on disease, the exact mechanisms by which diseases develop remain unclear. We currently investigate the correlation between CRGs and immune infiltrates and the immunological variability of different cuproptosis-related molecular clusters in UC patients. A RF model for 5 genes is considered the best machine learning model for detecting UC tissue subtypes and the predictive model for UC patients. In our study, we identify for the first time the role of cuproptosis in UC and reveal the unknown underlying molecular mechanisms of UC.

## Data Availability

The datasets supporting the conclusions of this article are available in the GEO website (https://www.ncbi.nlm.nih.gov/geo/), with the following data accession identififiers: GSE179285, GSE107597and GSE92415.
