# Correction to: Identification and immunological characterization of cuproptosis-related molecular clusters in ulcerative colitis

**DOI:** 10.1186/s12876-023-02894-1

**Published:** 2023-07-31

**Authors:** Yunfei Pu, Xianzhi Meng, Zhichen Zou

**Affiliations:** 1 The First Affiliated Hospital of Harbin Medical University, Harbin, Heilongjiang China; 2 Department of Minimally Invasive Biliary Surgery, The First Affiliated Hospital of Harbin Medical University, Harbin, 150000 Heilongjiang China

**Correction: BMC Gastroenterol 23, 221 (2023)**

10.1186/s12876-023-02831-2

Following publication of the original article [1], it was reported that Zhichen Zou should be the corresponding author instead of Xianzhi Meng. This Correction reflects the updated authorship, and the original article has been updated.
