# Correction: Identification and immunological characterization of cuproptosis-related molecular clusters in ulcerative colitis

**DOI:** 10.1186/s12876-024-03236-5

**Published:** 2024-05-29

**Authors:** Yunfei Pu, Xianzhi Meng, Zhichen Zou

**Affiliations:** 1 The First Affiliated Hospital of Harbin Medical University, Harbin, Heilongjiang, China; 2 Department of Minimally Invasive Biliary Surgery, The First Affiliated Hospital of Harbin Medical University, Harbin, Heilongjiang, 150000 China

**Correction:** ***BMC Gastroenterol*** **23, 221 (2023)**

10.1186/s12876-023-02831-2

Following publication of the original article and a connected Correction [1], it was reported by the authors that the corresponding authorship was erroneously switched from Xianzhi Meng to Zhichen Zou. This change has been undone and the corresponding authorship returned to Xianzhi Meng.

Furthermore, the authors have agreed at the suggestion of the Editors to remove the original reference 25 [2] appearing at the end of the first sentence of the ‘Materials’ section from the original article due to the lack of relevance to their study.

The original article has been updated.
